# Quantifying the persisting orphan-drug shortage public health crisis in the United States

**DOI:** 10.1080/20016689.2017.1269473

**Published:** 2016-12-23

**Authors:** Szymon Jarosławski, Chiraz Azaiez, Daria Korchagina, Mondher Toumi

**Affiliations:** ^a^ Public Health Department, EA 3279, Aix – Marseille University, France; ^b^ Mental Health and Public Health, Inserm U669, Maison de Solenn, Paris Cedex, France

**Keywords:** Orphan drugs, rare diseases, drug shortage, public health

## Abstract

**Background**: Orphan drugs (ODs) are pharmaceuticals manufactured for rare conditions that affect less than 200,000 people in the US. ODs are therefore produced in small quantities to meet sparse demand. Since 2010, OD shortages have become frequent, but no comprehensive, quantitative studies exist.

**Objective**: The objective of this study is to assess the rates of OD shortages per therapeutic class and their trends over time in the United States.

**Study design**: OD approvals were collected from publicly available information on the US Food and Drug Administration (FDA) website on 13 June 2016. Data on OD shortages were collected from the FDA and the American Society of Health-System Pharmacists (ASHP) websites. We reviewed the number of shortages per year and per therapeutic area. Multiple indications for the same drug were counted individually.

**Results**: Of 569 ODs approved, 50% were approved in the decade ending in 2015. Oncology was found to be the most represented therapeutic area (34% of all OD approvals), followed by endocrinology (11%). Shortage data were available from 2008. In total, there were 66 (12%) OD shortages, with an average shortage duration of 455.5 days. Shortages were observed mainly for oncology products (19 cases, 13% of oncology ODs) and endocrinology products (14 cases, 22% of endocrinology ODs)

**Conclusion**: Despite the FDA strategic plan for preventing and mitigating drug shortages (October 2013), remaining OD shortages still pose an enduring challenge to patient care, with a median shortage duration of almost 15 months. In many instances, ODs are the only available therapy for rare diseases, and OD shortages can lead to serious health deterioration and death. More research is needed to elucidate the causes of shortages and their impact on patients’ health.

## Introduction

The US Food and Drug Administration (FDA) defines a drug shortage as a situation in which the total supply of all clinically interchangeable versions of an FDA-regulated drug is inadequate to meet the current or projected demand at the patient level [1]. New and ongoing drug shortages are a serious public health issue in the US. New drug shortages reported per year rose dramatically from 70 in 2006 to a high of 267 in 2011 [2]. Active shortages have been persistent, and their cumulative number increased in the period from 2010 to 2014, crossing the 450 mark in 2012 [2]. Further, drug shortages may cost hospitals over $416 million a year [3].

Orphan drug (OD) shortages are of special concern, since they are used to treat rare diseases affecting less than 200,000 people in the US [4]. Moreover, due to a special period of market exclusivity granted to OD manufactures by the FDA, one company typically produces the drug’s entire supply. Consequently, manufacturing interruptions or discontinuances of ODs result in patients being deprived of the only available treatment. The case of a US biotech company Genzyme exemplifies this issue. When the company had to close its manufacturing plants due to quality issues, patients relying on the company’s OD suffered serious health consequences, including death [5]. In a suit against the company by a spouse of a deceased patient, the court said that public policy forbids the court to impose a duty of care on drug manufacturers and dismissed the argument that the company owed patients a duty of reasonable care [6]. Therefore, since courts alone cannot guarantee legal protection against OD shortages, systemic solutions that prevent and mitigate such shortages are urgently needed.

Previous work has identified OD shortages as a matter of public health concern and proposed solutions to mitigate such events [6]. It concluded that both US Congress and the FDA should not only aim to prevent orphan drug shortages, but find alternative, compensatory solutions for patients harmed by these shortages. However, there are no published quantitative studies on OD shortages showing the scale of this phenomenon. In this study, we sought to estimate quantitatively the extent of OD shortages in the US across various therapeutic areas, in order to inform such much-needed future policies.

## Methods

### Data sources

Data on OD shortages were extracted from the FDA (http://www.accessdata.fda.gov/scripts/drugshortages) and from the American Society of Health-System Pharmacists (ASHP) (http://www.ashp.org/menu/DrugShortages.aspx) websites from the beginning of data availability in 2008 to the day of data extraction on 13 June 2016. OD approvals by the FDA between 1983 and 16 June 2016 were collected from publicly available information on the FDA website (http://www.accessdata.fda.gov/scripts/opdlisting/oopd/index.cfm).

### Data analysis

Shortage data extracted from the FDA and ASHP were collected in an Excel sheet, including the name of the product, therapeutic category, availability of the product, status of the product, date of the beginning of shortage and duration of the shortage, and information on the type of the drug (biologic/small molecule). Duplicate information on drugs in shortage existing in both databases was removed. Multiple approvals of a drug in different indications were counted separately. Multiple shortages for a drug were counted separately.

OD shortages were categorized in three different statuses: currently in shortage (when the drug is in shortage in a given year); resolved (when the FDA Drug Shortages Staff determines that the market is covered, based on information from all manufacturers [7]); and discontinued by the FDA due to adverse events or other reasons, or by the companies due to commercial reasons [8]. Active shortages in a given year were counted if the shortage lasted at least one day in that year.

We reviewed the number of OD shortages per year, the therapeutic class, and the type of molecule (biologic or small).

## Results

**Figure 1.**
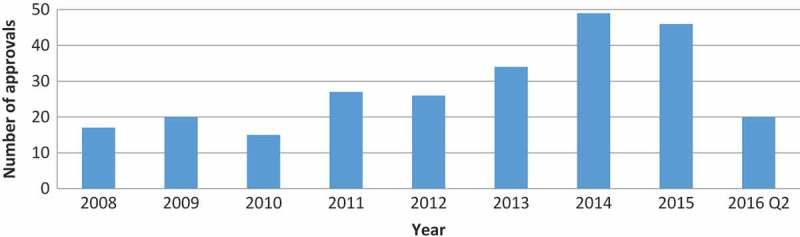
Number of new OD approvals per year.

Five hundred and sixty-nine ODs were approved by the FDA since the provision of the Orphan Drugs Act in 1983. However, data on OD shortages were available only for the period from January 2008 to June 2016, and yearly distribution of approvals is shown in Figure 1 only in this period. The distribution of therapeutic areas among all approved ODs is shown in Table 1. There were 232 (40.8%) biologic drugs.

**Table 1.** Number of OD approvals by therapeutic area.

	Rheumatology	Nephrology	Antidotes	Gastroenterology	Respiratory	Immunology	Others	Cardiovascular	Ophthalmology	Anti-infectives	Neurology	Hematology	Endocrinology	Oncology
Number of OD approvals	12	13	14	15	16	24	30	31	33	48	48	54	63	150
Percentage of all ODs	2%	2%	2%	3%	3%	4%	5%	5%	6%	8%	8%	9%	11%	26%

**Figure 2.**
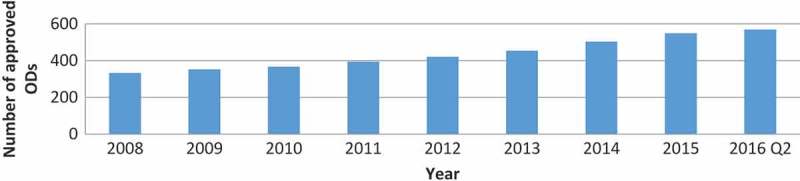
Cumulative number of FDA-approved ODs on the US market by year.

There were no shortage data disagreements between the FDA and ASHP databases, but the latter contained more data. Figure 2 shows the cumulative number of ODs approved on the US market in the period for which shortage data was available.

**Figure 3.**
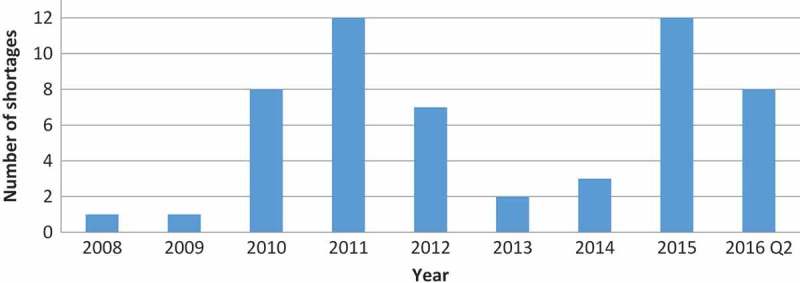
Yearly distribution of new OD shortages.

Altogether, there were 66 shortages, which constituted 12% of all ODs approved since 1983. Yearly distribution of new shortages is shown in Figure 3. There was no consistent trend in the number of new shortages in this period.

**Figure 4.**
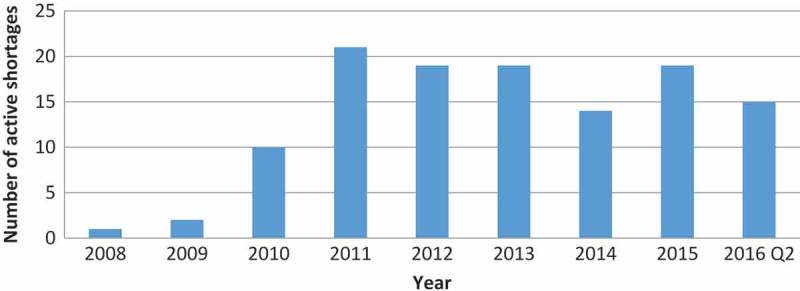
Number of active OD shortages in a given year (with a minimal shortage duration of one day).

**Figure 5.**
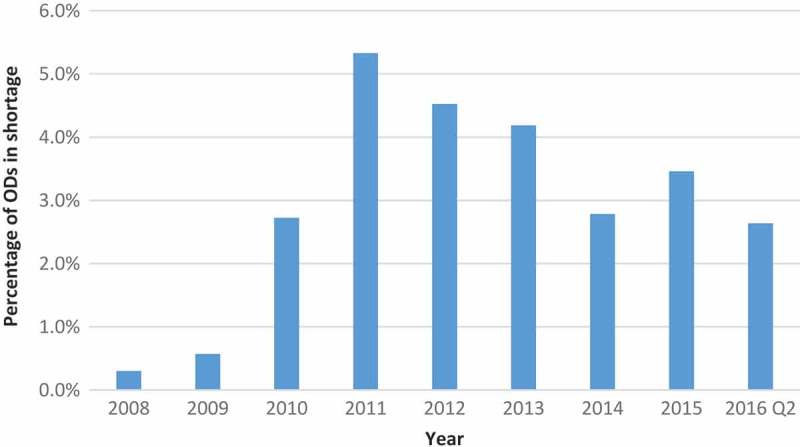
Percentage of ODs on the market that were in shortage in a given year for at least one day.

Because certain shortages lasted longer than one year, we also calculated the number of active OD shortages per year (Figure 4). Whereas the highest number of new OD shortages per year was 12 (in 2011 and 2015), the number of active shortages was as high as 21 in 2011. The proportion of ODs on the US market that were affected by shortage ranged from 0.3% in 2008 to 5.3% in 2011 (Figure 5).

**Figure 6.**
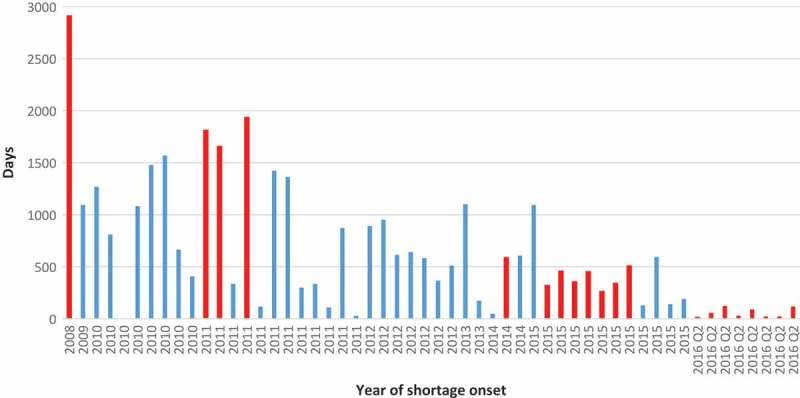
Duration of individual shortages starting in a given year. Note: Red bars indicateshortages unresolved at the date of data collection (13 June 2016).

The duration of individual shortages ranged from three to 2,912 days (Figure 6). The longest shortage began in 2008 and lasted until data collection day. This shortage was for ‘multi-vitamin infusion without vitamin K’, indicated for ‘prevention of vitamin deficiency and thromboembolic complications in people receiving home parenteral nutrition and warfarin-type anticoagulant therapy’. Median shortage duration for the entire data set was 455.5 days. However, this figure is likely to be underestimated, because the majority of shortages that commenced in 2015 and later were still unresolved on the day of data extraction.

In terms of therapeutic areas affected, shortages were observed mainly for oncology products (19 cases, 13% of oncology ODs) and endocrinology products (14 cases, 22% of endocrinology ODs) (Table 2). Antidotes and immunology products were not affected by shortages, and only one pulmonology product (6% of respiratory ODs) and two nephrology products (15% of nephrology ODs) were marked by shortages.

**Table 2.** Distribution of shortages among therapeutic areas.

	Antidotes	Anti-infectives	Cardiovascular	Endocrinology	Gastroenterology	Hematology	Immunology	Nephrology	Neurology	Oncology	Ophthalmology	Others	Respiratory	Rheumatology	Total
**Number of shortages**	0	3	4	14	3	4	0	2	5	19	3	4	1	4	66
**Number of ODs**	14	48	31	63	15	54	24	13	48	150	33	30	16	12	569
**Percentage of ODs affected by shortage**	0%	6%	13%	22%	20%	7%	0%	15%	10%	13%	9%	13%	6%	33%	12%

## Discussion

A rapid growth in OD approvals shown in our study has been previously reported [9,10]. The exact numbers differ slightly from those reported by Karst, due to methodological differences [10].

In terms of distribution of therapeutic areas of ODs, our findings largely confirm previous work. A study that looked at OD approvals in the US between 2001 and 2010 found that oncology was the most common therapeutic area in the sample, with 47% of approvals [9].

The increasing trend in drug shortages has been well documented [2,11,12]. ODs are particularly prone to shortages due to manufacturing difficulties [6,12]. However, our findings suggest that the number of new OD shortages per year is variable. Nevertheless, since the median duration of a shortage was nearly 15 months, the number of active OD shortages per year remained steadily high since 2011. The long duration of certain shortages is the most striking finding in our study.

However, because the number of new ODs on the market grew steadily in the study period, the percentage of ODs that experienced a shortage had a decreasing trend since the maximum of 5.3 % in 2011. Lack of data for remaining two quarters of the year 2016 makes it difficult to conclude if this trend will continue.

Whereas the FDA’s OD designation review should ensure that no alternative treatments exists for a given rare disease [13], we did not review the indications of ODs in shortage to confirm that they can result in serious health consequences due to lack of alternative treatments. This work should constitute a separate study.

In line with our finding that oncology was the most affected by OD shortages, shortage prevalence for oncology drugs in the US has been estimated at 35.8%, and 59–83% of US oncologists said that they faced shortages of cancer treatments [14,15]. However, in the former study, OD status was associated with lower shortage prevalence, suggesting that rare cancers are less affected by shortages than the more common cancers. Nevertheless, it is likely that oncology ODs lack alternative treatment options, and thus their shortages pose a serious health threat. This requires further research. We could not identify published data on shortage prevalence in other therapeutic areas.

One cited reason for frequent OD shortages is that the majority of these drugs are biologics [16] and are delivered as sterile injectables [11]. Both of these characteristics make them prone to market recalls due to quality issues [2,6,12]. However, in our sample, biologics constituted only about 40% of ODs.

Further, given that OD approval is expedited and less information may be available for the regulator, manufacturing protocols may contain inherent errors or inadequacies that lead to quality issues and recalls. That’s why the FDA needs to balance the risk to product quality and expedited availability for patients [17].

Further, since OD production is commonly limited to a single source, this exaggerates manufacturing problems [12]. In the lack of alternative suppliers, the shortages cannot be supplemented from different manufacturers. Therefore, market exclusivity which OD manufacturers enjoy has encouraged the development of new treatments for rare diseases, but it has also made these drugs more vulnerable to shortages [12].

There are several recent policies that both the US Congress and the FDA have developed to address the drug shortage crisis. In 2012, the FDA enacted the Safety and Innovation Act (FDASIA) that requires manufacturers to report to the FDA discontinuation or disruption of supply of life-supporting, life-sustaining drugs, or drugs for debilitating conditions [18]. As a result of the FDASIA, the FDA developed a Strategic Plan for Preventing and Mitigating Drug Shortages in 2013, which stated that preventing drug shortages remains a top priority for the FDA [19]. Early notification of the FDA by manufacturers about possible interruptions was the cornerstone of the plan, which also assumed strengthening the FDA’s capacity to respond to notifications, as well as developing long-term prevention strategies. Also, in 2011, President Obama signed an Executive Order that increased the FDA’s jurisdiction in preventing and reducing drug shortages of life-saving drugs. It granted the FDA broader reporting of discontinuances, expedited regulatory review, and the ability to report drug profiteering activities to the Department of Justice [6].

Other actions against shortages include the FDA’s consent decrees against manufacturers with persisting manufacturing quality issues, which result in very high fines [6,20].

Specifically, if the FDA believes an OD maker cannot produce enough drugs to meet patient need, the Agency can offer the company information on how the company can produce sufficient orphan drug quantities within a reasonable time, or the drug maker may agree to allow the FDA to approve competitor drugs before the company’s market exclusivity expires [6,21]. Otherwise, it can withdraw the drug’s exclusive approval permanently.

However, these efforts have not significantly reduced drug shortages [22]. Moreover, some of these actions are inadequate for OD shortages. For example, while the FDA can accelerate review of manufacturing lines or seek manufacturers of similar products to increase production during shortages, ODs are usually produced by a single manufacturer. Three solutions to mitigate and prevent OD shortages have been proposed recently [6]:

- the FDA should require OD companies to create redundant or back-up manufacturing processes and systems;
- the FDA should develop an OD stockpile repository programme (it should require the OD maker to manufacture excess drugs every year);
- Congress should propose and pass legislation that would allow the FDA to establish an OD compensation fund that would provide monetary relief for patients harmed by OD shortages.

The first two solutions will likely encounter resistance from the industry, as they may lead to increased costs for OD makers.

## Conclusions

To our knowledge, this is the first quantitative study that analyses comprehensive OD shortage data in the US. The most striking finding is that the OD shortages tend to be long-term, with a median duration of almost 15 months. This area requires further research as to the causes of long-term OD shortages that can lead to serious health consequences, including deterioration of health and death. This research is needed to inform new, more effective public health policies to tackle the OD shortage crisis.
